# From the archives: Cold acclimation by plastid translation, metabolic allocation, and plant defense, and functions of a BiP–calreticulin complex

**DOI:** 10.1093/plcell/koad041

**Published:** 2023-02-15

**Authors:** Lucas Frungillo

**Affiliations:** Assistant Features Editor, The Plant Cell, American Society of Plant Biologists, USA; Institute of Molecular Plant Sciences, School of Biological Sciences, University of Edinburgh, UK

## May 2022: Cold acclimation by plastid translation

**Figure 1.**
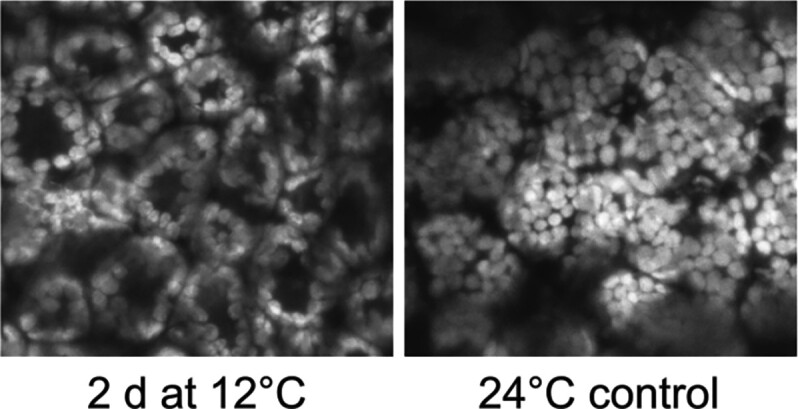
Cold-induced chloroplast movements. A top–down view of chloroplasts in mesophyll cells of acclimating and control plants grown for 2 d after cold shift. Reprinted from Gao et al. (2022), Fig. 1.

Cold stress is a severe environmental constraint to plant growth and development. Low temperatures trigger several physiological and structural changes in chloroplasts, including light avoidance-like movements (see Fig. 1), photoinhibition of PSII, and a consequent burst in reactive oxygen species production at the photosynthetic electron transport chain. These observations suggest that chloroplast homeostasis plays a central role in regulating responses to cold. Gao et al. showed that translational activity in chloroplasts mediates tobacco acclimation to low temperatures. While translation is severely compromised by cold shock in procaryotes (Weber and Marahiel 2003), the authors showed that cold stress triggered only a partial reduction in chloroplast translation initiation in plants. Moreover, low temperatures triggered transient changes in ribosome distribution on chloroplast reading frames, causing translation adjustments of nonessential photosynthetic subunits. Reverse genetics revealed that chloroplast translational regulation of the nonessential subunit of the cytochrome b6f complex, PetL, is required for cold tolerance. It is tempting to speculate whether translational adjustments evolved as an energy-efficient alternative to overcome inherent overall deacceleration of metabolic fluxes during low temperatures.

## May 2018: Metabolic allocation and plant defense

Diterpenoids are a class of natural compounds with strong antimicrobial activity. In rice, *ent*- and *syn*-copalyl diphosphate (CPP) intermediates define opposing branches of the diterpenoid biosynthetic pathway. Despite diterpenoid structural diversity, the antimicrobial activity of rice diterpenoids had been characterized mainly against adapted fungal blast *Magnaporthe oryzae* in vitro (Peters 2006). Lu et al (2018) used an innovative metabolic allocation strategy to uncover the role of different diterpenoid branches in rice immune responses. By taking advantage of its well-documented biosynthetic pathway, the authors used reverse genetics to manipulate endogenous levels of either *ent*-CPP- or *syn*-CPP-derived diterpenoids. While suppression of the *ent*-CPP pathway rendered rice plants more susceptible to *M. oryzae* and bacterial leaf blight *Xanthomonas oryzae*, mutation of *syn*-CPP synthase specifically enhanced resistance against *X. oryzae* and non-host *Magnaporthe poae*. Thus, despite showing similar antimicrobial activity in vitro, diterpenoids selectively contribute to immune responses in planta. Also, this study provides evidence that manipulation of metabolic partitioning is a viable strategy to infer the functional roles of secondary metabolites.

## May 1998: Functions of a BiP–calreticulin complex

The endoplasmic reticulum (ER) plays a central role in the biosynthesis and transport of proteins in cells. At the ER lumen, molecular chaperones aid newly synthesized polypeptides to fold into 3D structures and gain functional activity. The BINDING IMMUNOGLOBULIN PROTEIN (BiP) and CALRETICULIN (CALR) are evolutionarily conserved chaperones present in ER lumen, but the interplay between BiP and CALR was previously unknown. Crofts et al (1998) showed that the chaperone BiP forms an abundant, ATP-sensitive protein complex with CARL in the ER lumen. Co-immunoprecipitation assays showed that different BiP motifs bind to CARL and the model assembly-defective polypeptide, phaseolin. Interestingly, co-immunoprecipitation of phaseolin and CARL was not observed, suggesting that different pools of BiP coexist in the ER. Further genetic and biochemical assays indicated that BiP availability is the limiting factor in the BiP–CARL complex formation and that BiP–CARL complex formation is independent of cellular stress. The authors, therefore, hypothesized that BiP–CARL complex is involved in ER homeostasis, whereas the chaperone activity of free BiP plays a role in stress responses.

In a follow-on study, while BiP overexpression only mildly increased protein levels, BiP degradation products were detected in tobacco overexpressing lines (Leborgne-Castel et al. 1999). These results indicate that BiP protein levels feedback regulates its own proteolysis to prevent BiP overaccumulation in the ER. Because BiP is essential for cell viability, it is tempting to speculate that the BiP–CALR complex represents a strategy to titrate free BiP availability or its retention in the ER. The investigation of mechanisms underlying BiP–CARL complex stability, potentially posttranslational modifications targeting BiP and CARL, may prove fruitful to understand BiP homeostasis in ER.
